# Whole-Brain Mapping of Neuronal Activity in the Learned Helplessness Model of Depression

**DOI:** 10.3389/fncir.2016.00003

**Published:** 2016-02-03

**Authors:** Yongsoo Kim, Zinaida Perova, Martine M. Mirrione, Kith Pradhan, Fritz A. Henn, Stephen Shea, Pavel Osten, Bo Li

**Affiliations:** ^1^Cold Spring Harbor LaboratoryCold Spring Harbor, NY, USA; ^2^Department of Neural and Behavioral Sciences, College of Medicine, Penn State UniversityHershey, PA, USA; ^3^MRC Laboratory of Molecular BiologyCambridge, UK; ^4^Medical Department, Brookhaven National LaboratoryUpton, NY, USA; ^5^Department of Biomedical Sciences, Quinnipiac UniversityHamden, CT, USA; ^6^Psychiatry Department, Mount Sinai School of MedicineNew York, NY, USA

**Keywords:** *C-fos* expression, serial two-photon tomography, Positron-emission tomography, learned helplessness, depression

## Abstract

Some individuals are resilient, whereas others succumb to despair in repeated stressful situations. The neurobiological mechanisms underlying such divergent behavioral responses remain unclear. Here, we employed an automated method for mapping neuronal activity in search of signatures of stress responses in the entire mouse brain. We used serial two-photon tomography to detect expression of c-FosGFP – a marker of neuronal activation – in *c-fosGFP* transgenic mice subjected to the learned helplessness (LH) procedure, a widely used model of stress-induced depression-like phenotype in laboratory animals. We found that mice showing “helpless” behavior had an overall brain-wide reduction in the level of neuronal activation compared with mice showing “resilient” behavior, with the exception of a few brain areas, including the locus coeruleus, that were more activated in the helpless mice. In addition, the helpless mice showed a strong trend of having higher similarity in whole-brain activity profile among individuals, suggesting that helplessness is represented by a more stereotypic brain-wide activation pattern. This latter effect was confirmed in rats subjected to the LH procedure, using 2-deoxy-2[18F]fluoro-D-glucose positron emission tomography to assess neural activity. Our findings reveal distinct brain activity markings that correlate with adaptive and maladaptive behavioral responses to stress, and provide a framework for further studies investigating the contribution of specific brain regions to maladaptive stress responses.

## Introduction

Coping with various kinds of environmental stress is a fundamental brain function. However, persistent stress can often lead to mental disorders, including depression (Franklin et al., 2012). A number of animal models have been developed for studying the mechanisms of depression (Krishnan and Nestler, 2008). The learned helplessness (LH) procedure has been extensively used to produce stress-induced depression-like behavior in rodents (Abramson et al., 1978; Vollmayr and Henn, 2003), and has proved useful in preclinical studies (Vollmayr and Henn, 2003) as well as in studies investigating the neurobiological processes that may be involved in the pathogenesis of stress-induced depression (Li et al., 2011; Wang et al., 2014; Perova et al., 2015). Previous studies based on this model have explored brain activity measurements related to distinct behavioral phenotypes, which led to the discovery of several behaviorally relevant circuit changes (Mirrione et al., 2014; Wang et al., 2014; Perova et al., 2015). However, most of these studies have focused on selected brain regions and thus might have missed additional brain regions or functional features critical for the expression of the stress-induced depression-like behavior.

In this study we examined whole-brain activity patterns using automated unbiased mapping at single-cell resolution (Kim et al., 2015) in mice subjected to the LH procedure. Specifically, we used the expression of green fluorescent protein-tagged immediate early gene product c-Fos (c-FosGFP) in the c-*fosGFP* transgenic mice (Barth, 2004; Reijmers et al., 2007) as an indicator of neuronal activation, which was imaged across the entire brain with serial two-photon tomography (STPT; Ragan et al., 2012). As an alternative approach, we also used 2-deoxy-2[18F]fluoro-D-glucose positron emission tomography (18FDG-PET; Mirrione et al., 2014) to assess neural activity in rats subjected to the LH procedure. We identified a list of brain regions that show differential activity in helpless versus resilient animals. In addition, we uncovered abnormally stereotypic brain activity in helpless animals. Our study demonstrates the utility of inspecting brain-wide activity patterns for revealing circuits participating in specific behaviors, and supports the view that defining neuronal circuits underlying stress-induced depression-like behavior in animal models can help identify new targets for the treatment of depression.

## Results

### Behavioral Responses of Mice to the LH Procedure

**FIGURE 1:**
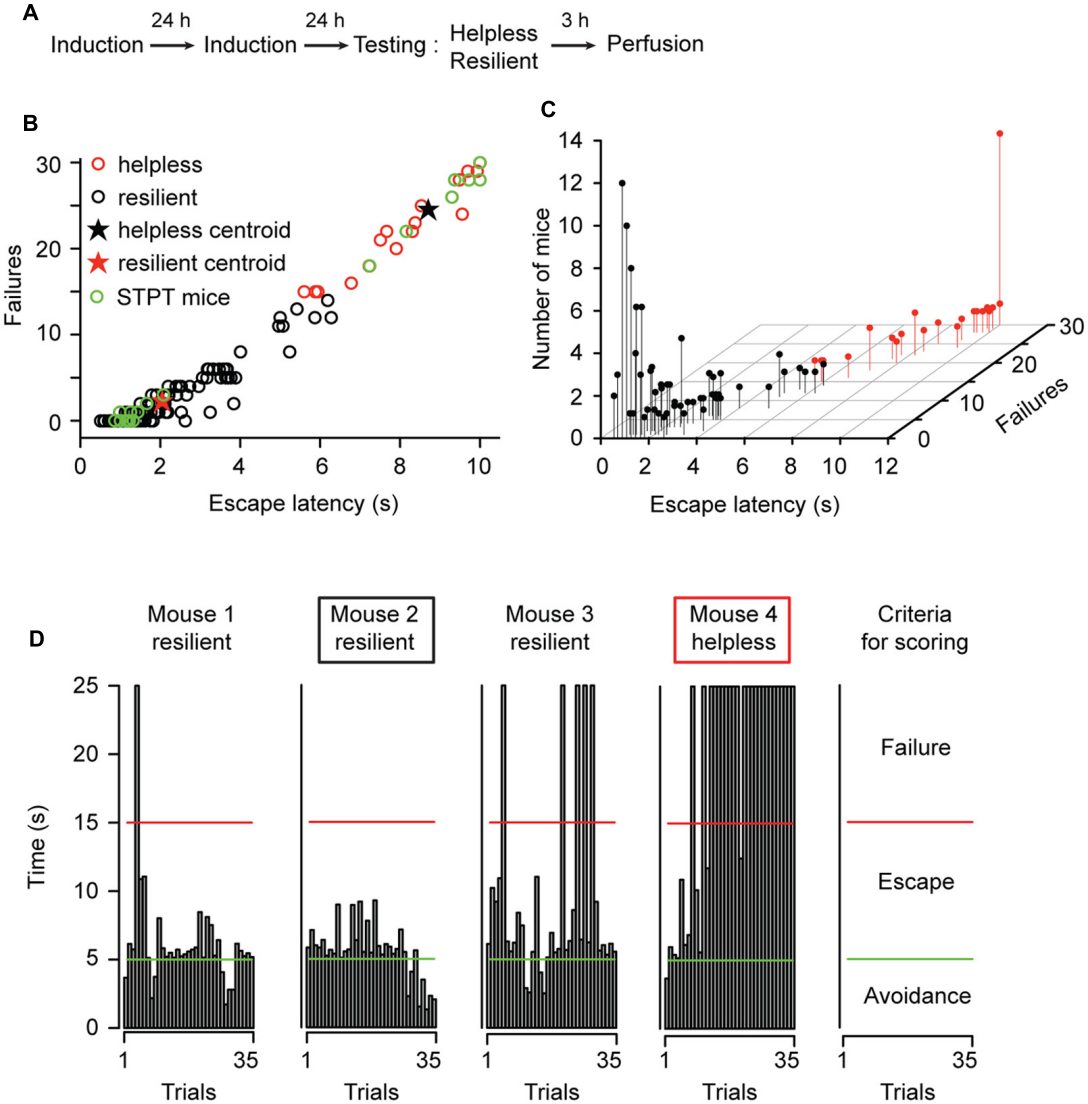
**Characterization of behavioral responses in the learned helplessness procedure. (A)** A schematic of the experimental procedure. **(B)** A graphic representation of the behavioral measurements (escape latency and failures) for all mice (*n* = 144) used in this study. A *k*-means cluster analysis defined two distinct clusters, the “helpless” and “resilient.” Mice chosen for further STPT imaging analysis is highlighted with green dots (STPT mice). **(C)** A 3D scatterplot of the same data shown in **(B)**. The position of each dot along the z-axis represents the number of mice showing a specific set of behavioral parameters. **(D)** Behavioral measurements from four representative mice tested in parallel. Each graph shows the time (y-axis) an animal took to finish each of the 35 trials (x-axis) in a testing session (see Materials and Methods). Trials that were ended within 5 s (bars under the green line) represent avoidance; trials that were ended within 15 s (bars under the red line) represent escape. The remaining trials represent failure. The rightmost panel summarizes the criteria for scoring. Mouse 2 and mouse 4 (marked by a black box and red box, respectively) were mice with extreme behavioral measurements, and they were chosen for STPT imaging (total 11 pairs, with one helpless and one resilient in each pair).

To mimic environmental stressors associated with mood disorders, we used the LH procedure in which animals were subjected to periods of inescapable and unpredictable foot shocks (**Figures 1A–C**; and see Materials and Methods; Chourbaji et al., 2005; Li et al., 2011; Perova et al., 2015). To achieve a subsequent detection of neuronal activity related to distinct behavioral responses, we used the c-*fosGFP* transgenic mice expressing c-FosGFP under the control of a *c-fos* promoter (Barth, 2004; Reijmers et al., 2007; Wang et al., 2014; Kim et al., 2015; Perova et al., 2015). The expression of the c-*fosGFP* transgene has been previously validated to faithfully represent endogenous *c-fos* expression (Kim et al., 2015). Similar to wild-type mice (Wang et al., 2014; Perova et al., 2015), approximately 22% (32 of 144) of the c-*fosGFP* mice showed helplessness (**Figures 1B,C**), a depression-like phenotype whereby animals show reduced escape from escapable foot shocks (Maier, 1984; Chourbaji et al., 2005; Li et al., 2011; Perova et al., 2015); the rest of the animals were resilient. Separation of mice into resilient and helpless groups was based on a *k*-means cluster analysis using performance indices – escape latency and number of failures in a testing session – as parameters for classification (Perova et al., 2015; **Figure 1**; and see Materials and Methods). The helpless mice showed significantly more failures and longer escape latencies than the resilient mice [*W*_(144)_ = 3584, *P* = 2.2 × 10^-16^, and *W*_(144)_ = 3579, *P* = 2.2 × 10^-16^, two-tailed Mann–Whitney test].

### Whole-Brain Activity Mapping in Mice Subjected to the LH Procedure

**FIGURE 2:**
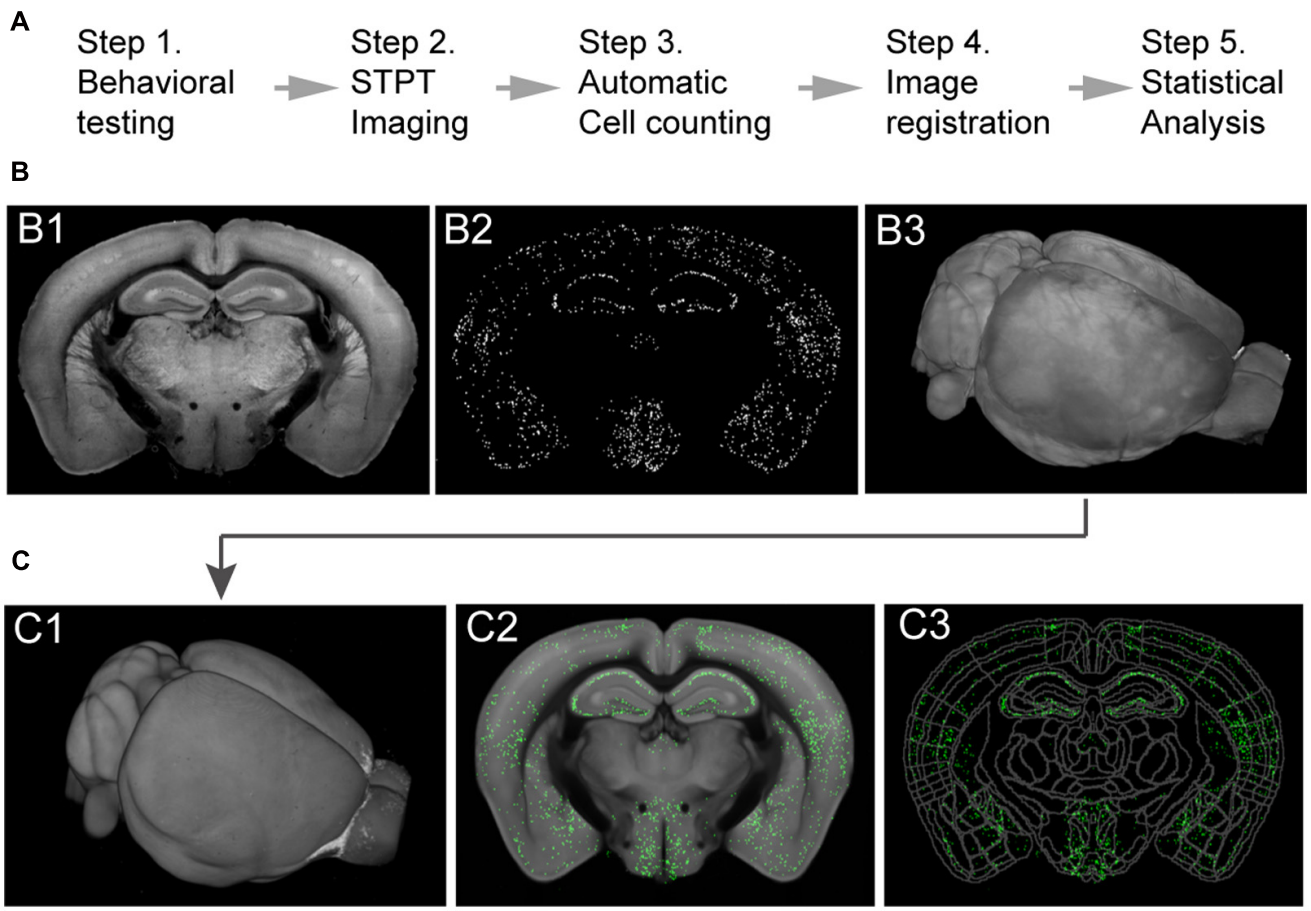
**Imaging whole-brain c-FosGFP in the *c-FosGFP* mice. (A)** A schematic of the cFos-GFP whole-brain data processing pipeline. (**B**; B1) A 2D-image of one sample brain taken with STPT. (B2) Machine learning algorithm-based c-FosGFP detection from the image shown in B1. (B3) 3D-reconstruction of the sample brain from serial 2D-images, including the one shown in B1. (**C**; C1) The sample brain is registered to a reference STPT brain before statistical analysis. (C2) The c-FosGFP signal transformed onto the reference brain. (C3) The reference brain is equipped with Allen Reference Brain anatomical labeling (http://atlas.brain-map.org).

**FIGURE 3:**
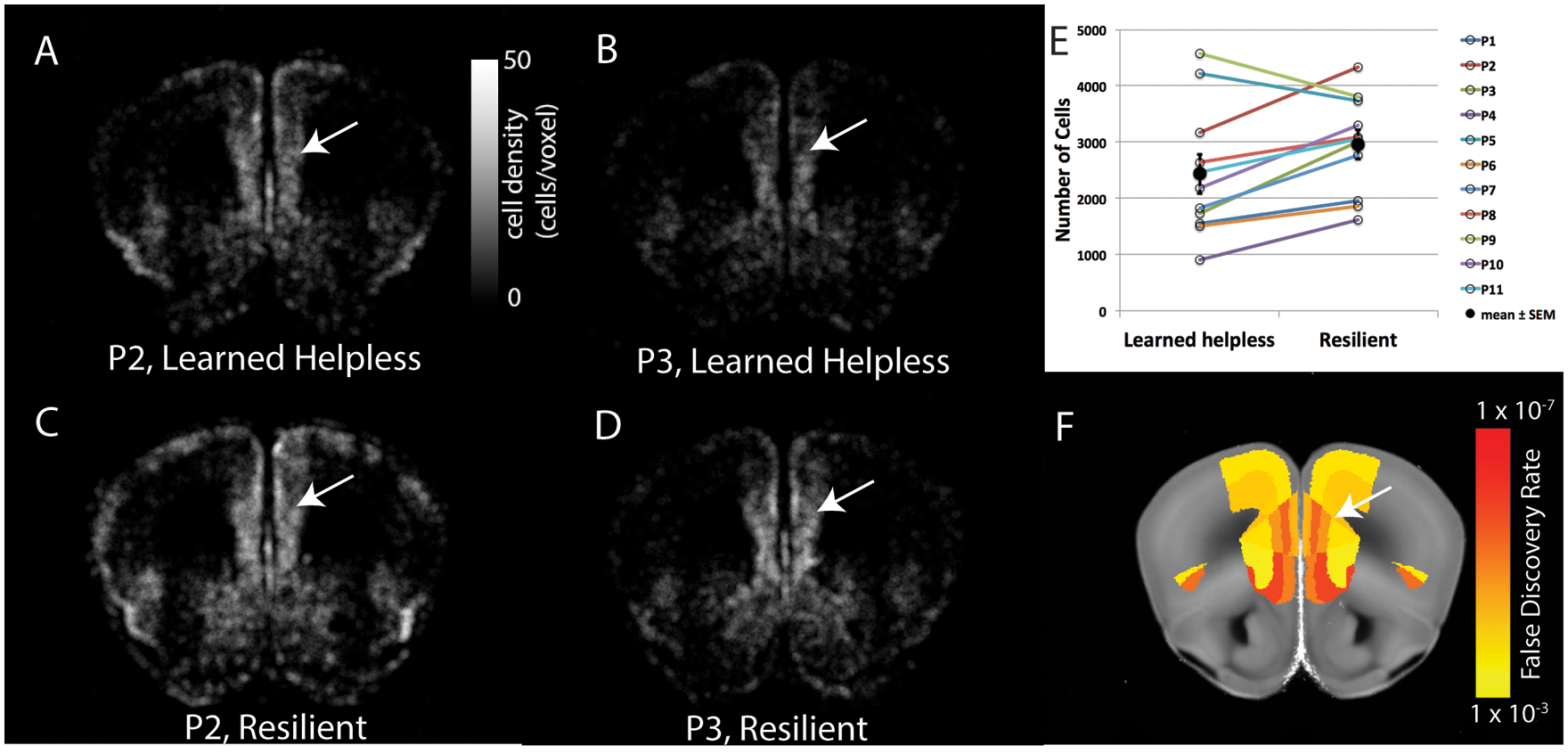
**Pair-wise comparison of brain cFos-GFP expression between the helpless and the resilient mice. (A–D)** The density of cFos-GFP^+^ cells in evenly spaced and overlapping sphere voxel (100 μm diameter) in a coronal brain section containing the prelimbic cortex, for two pairs of mice: P2 (**A**, helpless, **C**, resilient) and P3 (**B**, helpless, **D**, resilient). Heat map represents cFos-GFP cell counts per voxel. White arrows denote the prelimbic cortex. **(E)** Quantification of the number of cFos-GFP^+^ cells in the prelimbic cortex for the 11 pairs of mice. Filled circles and associated bars represent mean ± SEM. **(F)** The statistical results of pair-wise comparison for this brain section after multiple comparison correction by false discovery rate (FDR). Heat map represents FDR adjusted *P*-values. White arrow denotes the prelimbic cortex.

To facilitate the identification of neural changes potentially responsible for helplessness or resilience, in subsequent imaging experiments we used mice in each cohort that displayed the most extreme behavioral phenotypes: helpless mice that showed the most failures and longest escape latency, and resilient mice that had the least failures and shortest escape latency (**Figures 1B,D**). In addition, to control for potential fluctuations in c-FosGFP expression that are caused by non-specific factors, such as the time and date when c-FosGFP is examined, we collected pairs of mice for imaging, each pair containing a resilient mouse and a helpless one that had undergone the entire experimental procedure in parallel (**Figures 2** and **3**, see Materials and Methods; *N* = 11 pairs, one resilient and one helpless mouse per pair).

The mice were transcardially perfused 3 h after the LH testing session to allow the behaviorally driven c-FosGFP expression reach its maximal level (Kim et al., 2015; see Materials and Methods). The brains were collected and processed according to a previously established protocol (Kim et al., 2015), and subsequently imaged at cellular resolution by STPT (Ragan et al., 2012). Whole-brain c-FosGFP expression profile, representing the LH-induced brain activation in the helpless and resilient mice, was extracted and analyzed using previously established algorithms (Kim et al., 2015; **Figure 2**, see Materials and Methods).

**FIGURE 4:**
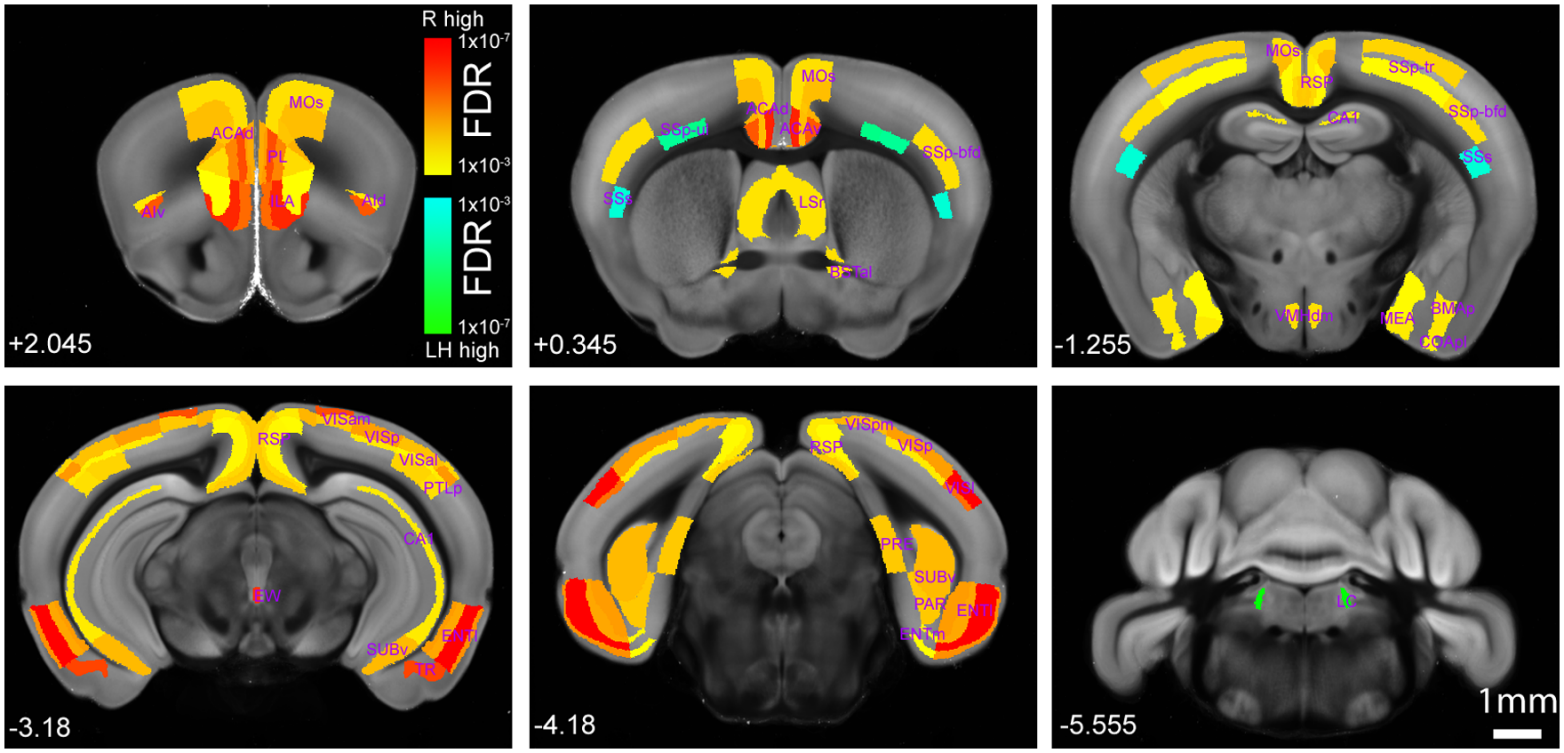
**Brain-wide comparison between the helpless and resilient mice.** Hot or cold colors represent anatomical regions of interest (ROI) with significantly higher activity in the resilient group (“R high”) or the learned helpless group (“LH high”), respectively, when comparisons are made between the two groups. Heat maps represent FDR *q*-values corrected for multiple comparisons after individual *P*-value in each brain region was computed. Numbers at the bottom denote coordinates relative to Bregma. A full dataset and the raw data containing cell counting in each anatomical area can be found in **Supplementary Tables S1** and **S2**. Please also see **Table 1**. The Interactive Allen Reference atlas with anatomical annotations can be found in http://atlas.brain-map.org.

**FIGURE 5:**
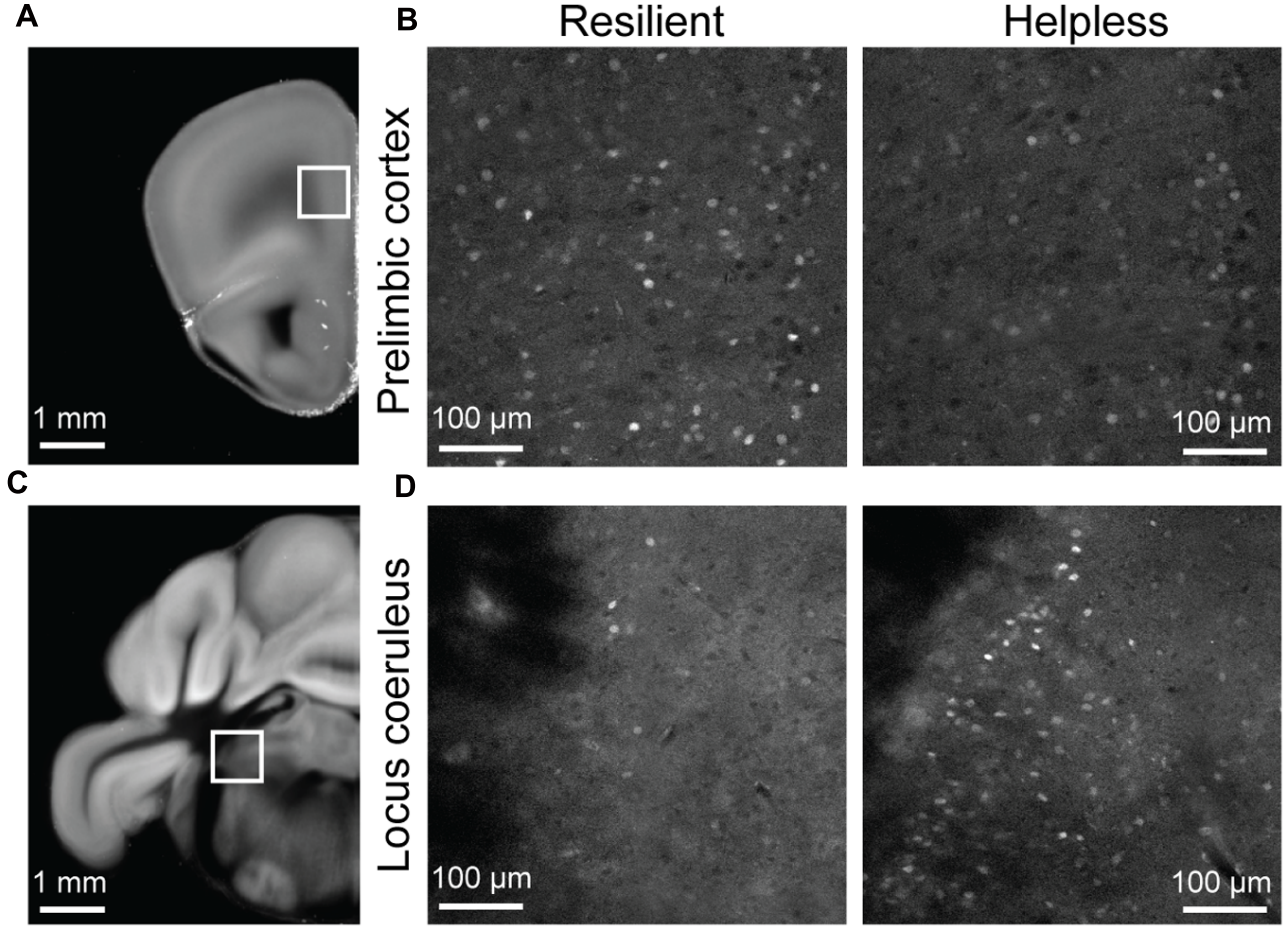
**Raw STPT data from selected regions of the *c-fosGFP* mice. (A)** A low magnification image of a brain section containing the prelimbic cortex (PL; boxed area). **(B)** High magnification images of the PL in a resilient mouse (left) and a helpless mouse (right). **(C)** A low magnification image of a brain section containing the locus coeruleus (LC; boxed area). **(D)** High magnification images of the LC in a resilient mouse (left) and a helpless mouse (right).

Overall, the helpless mice showed significantly lower levels of activity than the resilient mice in many brain areas, including several cortical and subcortical regions (**Figures 4** and **5**, **Table 1**, **Supplementary Tables S1** and **S2**). More specifically, we found that the helpless group had lower activation than the resilient group in high cognitive cortical areas including the medial prefrontal (PL, ILA, ORBm), AId, AIv, ACA, RSP, and PTLp (**Figures 4** and **5**, **Table 1**, **Supplementary Tables S1** and **S2**; see **Table 1** and **Supplementary Table S2** for abbreviations), many of which have been implicated in mood or anxiety disorders (Myers-Schulz and Koenigs, 2012; Price and Drevets, 2012). This result was corroborated for selected brain areas by an independent experiment in which endogenous c-Fos expression in the medial prefrontal cortex (mPFC) in mice was assessed by immunohistochemistry (**Supplementary Figure S1**).

**Table 1:** Selected brain areas showing differential activity in helpless versus resilient animals.

Parent regions	Acronym	Full name	FDR *q*-value
Isocortex	MOs	Secondary motor area	2.80E-03
	VIS	Visual areas	7.85E-05
	ACA	Anterior cingulate area	5.31E-03
	PL	Prelimbic area	1.67E-04
	ILA	Infralimbic area	3.96E-05
	ORBm	Orbital area, medial part	2.49E-03
	AId	Agranular insular area, dorsal part	5.29E-03
	AIv	Agranular insular area, ventral part	3.56E-03
	RSP	Retrosplenial area	4.02E-03
	PTLp	Posterior parietal association areas	3.87E-03
Hippocampal formation	CA1	Field CA1	1.18E-03
	CA2	Field CA2	4.43E-05
	ENTI	Entorhinal area, lateral part	4.62E-07
	PAR	Parasubiculum	5.29E-03
	PRE	Presubiculum	3.44E-03
	SUBv	Subiculum, ventral part	2.33E-03
Cortical Subplate	BLA	Basolateral amygdalar nucleus	5.93E-03
	BMAp	Basomedial amygdalar nucleus, posterior part	5.82E-03
Striatum ventral region	OT	Olfactory tubercle	8.65E-03
Lateral septum complex	LS	Lateral septal nucleus	6.83E-03
Striatum-like amygdala nuclei	AAA	Anterior amygdalar area	7.56E-03
	MEA	Medial amygdalar nucleus	5.29E-03
Pallidum	BSTal	Bed nuclei of the stria terminalis, anterior division, anterolateral area	5.17E-03
Hypothalamus	VMHdm	Ventromedial hypothalamic nucleus, dorsomedial part	4.96E-03
Midbrain	EW	Edinger-Westphal Nucleus	1.65E-05
Hind brain	LC	Locus ceruleus	1.55E-04

*The brain areas are arranged based on the Allen Brain Atlas ontological order. All listed brain areas had lower activity in helpless mice than resilient mice, with the exception of the LC (highlighted in purple), which showed higher activity in helpless mice than resilient mice (see also **Supplementary Tables S1** and **S2** for full data).*

The helpless group also showed lower activation in the lateral septum (LS) and olfactory tubercle (OT), which are involved in reward processing (Sheehan et al., 2004; Ikemoto, 2010; **Figure 4**, **Table 1**, **Supplementary Tables S1** and **S2**); the amygdala and extended amygdala regions (BLA, BMAp, AAA, BSTal, and MEA), which are critical for processing emotion and behavioral motivation (**Figure 4**, **Table 1**, **Supplementary Tables S1** and **S2**); the hypothalamic area (VMHdm), which is important for defensive behavior (Wang et al., 2015); the midbrain area (EW), which is implicated in stress coping (Kozicz et al., 2011; **Figure 4**, **Table 1**, **Supplementary Tables S1** and **S2**); and, lastly, the hippocampal regions (CA1, CA2, ENTl, PAR, PRE, SUBv), which are implicated in learning and memory, cognitive function, and emotional responses (Fanselow and Dong, 2010; **Figure 4**, **Table 1**, **Supplementary Tables S1** and **S2**).

Notably, our brain-wide analysis revealed that the locus coeruleus (LC) is the only subcortical area that had significantly enhanced activation in helpless compared with resilient mice (**Figures 4** and **5**, **Table 1**, **Supplementary Tables S1** and **S2**). This finding may be of particular clinical significance given the important role attributed to the noradrenergic system in stress response, and in the pathogenesis and treatment of depression (Krishnan and Nestler, 2010; Bradley and Lenox-Smith, 2013; Gold, 2015).

### Stereotypic Brain Activity Profile in Animals showing Helplessness

**FIGURE 6:**
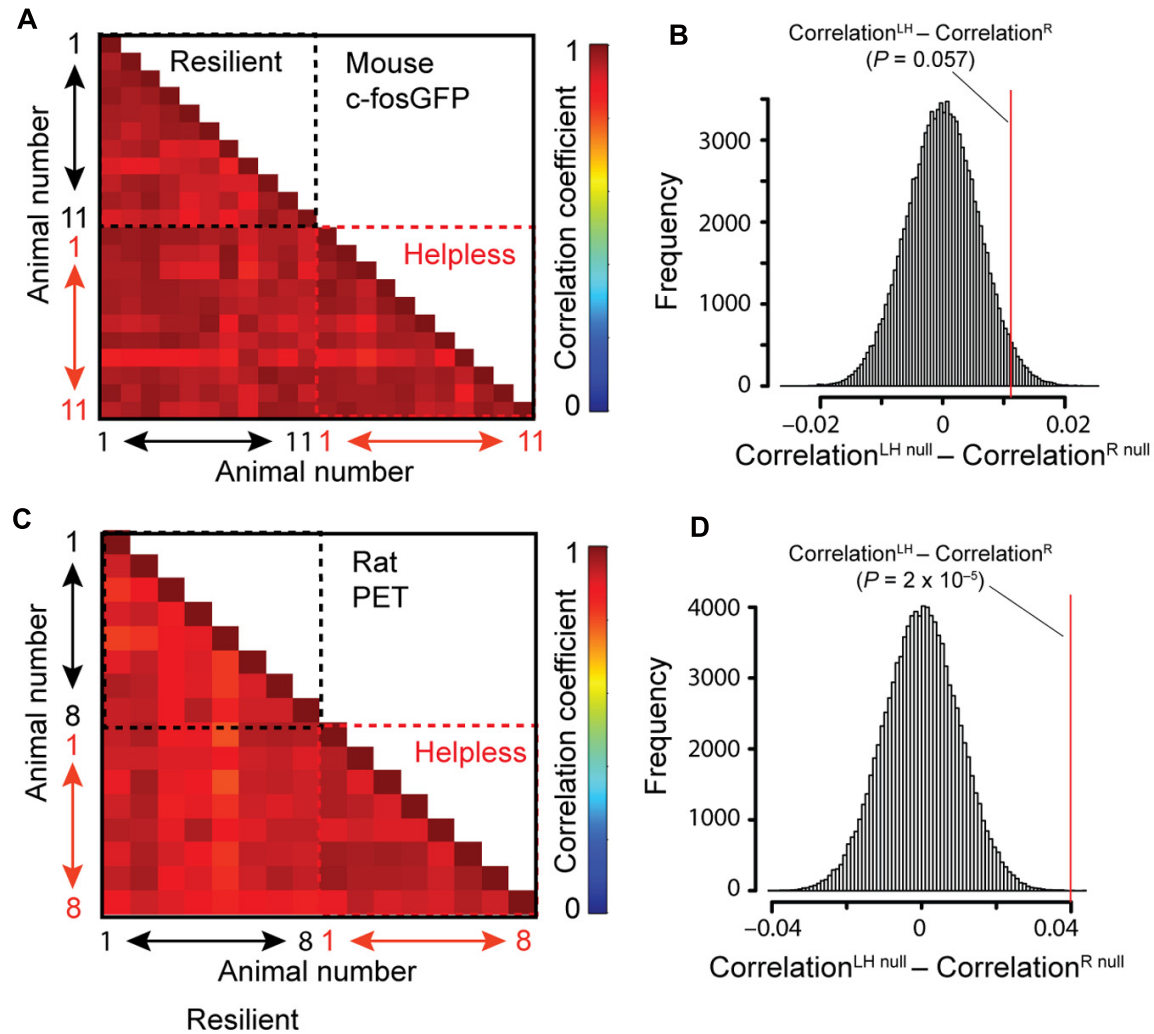
**Correlation between individual animals in brain-wide activity. (A,B)** Correlation analysis of c-FosGFP signal from the *c-fosGFP* mice. **(A)** Correlation coefficient for each pair of animals is displayed in a pairwise correlation matrix. **(B)** Result of the bootstrap test (see Materials and Methods for details). LH, learned helpless; R, resilient. Red line denotes the location in the null distribution (Correlation^LHnull^ – Correlation^Rnull^) where the difference between the actual LH and R groups (Correlation^LH^ – Correlation^R^) fell (*P*-value resulted from a two-sided test). **(C,D)** Same as **(A,B)**, except that correlation analysis was performed on PET data from rats.

The whole-brain imaging dataset provides a unique opportunity to examine whether the helpless group can be distinguished from the resilient one on the basis of brain-wide activity profile. To this end, we extracted the c-FosGFP count for all anatomical structures across the entire brain in each animal. To compare the global brain activity patterns across individuals, we computed the correlations between pairs of animals in their areal c-FosGFP counts. Interestingly, we found that mice in the helpless group showed a strong trend toward higher correlations than those in the resilient group (*P* = 0.057 by a two-sided bootstrap test; see Materials and Methods; **Figures 6A,B**), suggesting that helpless mice have increased similarity among them in their brain-wide activity patterns compared with resilient mice.

To investigate this result independently, we assessed brain metabolic activity by 18FDG-PET (Mirrione et al., 2014) in rats subjected to the LH procedure (see Materials and Methods). We found significantly higher correlations between individuals of the helpless group than those of the resilient group in brain-wide activity (*P* = 2 × 10^-5^; two-sided bootstrap test (see Materials and Methods); **Figures 6C,D**). Due to limited spatial resolution of PET imaging, this method could not be used to verify the brain regional differences between helpless and resilient animals identified based on STPT in the c-*fosGFP* transgenic mice. Together, these results indicate a characteristic, stereotypic brain-wide activity pattern in helpless animals that is distinct from the more heterogeneous brain activity patterns among resilient animals.

## Discussion

When confronted with stress, most individuals are resilient whereas others are prone to developing mood disorders. The brain mechanisms of such divergent behavioral responses remain poorly understood. One animal model that has been widely used for the study of neural changes underlying behavioral phenotypes related to mood disorders is the LH paradigm (Abramson et al., 1978; Vollmayr and Henn, 2003; Mirrione et al., 2014; Wang et al., 2014; Perova et al., 2015). In this study we provide a global view about how neural activity associated with helpless behavior is different from that associated with resilient behavior. In particular, by using unbiased and whole-brain imaging techniques, we uncover a number of cortical and subcortical brain structures that have lower activity in the animals showing helplessness than in those showing resilience following the LH procedure. We also identified the LC as the sole subcortical area that had enhanced activity in helpless animals compared with resilient ones.

Some of the brain areas identified in this study – such as areas in the mPFC, hippocampus, and amygdala – have been previously implicated in clinical depression or depression-like behavior in animal models (Drevets et al., 2008; Russo and Nestler, 2013; Wang et al., 2014; Perova et al., 2015). Consistent with our results (see **Table 1**), previous studies in which a small number of brain areas were examined (Steciuk et al., 1999; Huang et al., 2004) showed that the lateral septal nucleus (LS) and mPFC have lower c-Fos expression in helpless animals than in resilient ones. In addition, increased expression of c-Fos in the LC has also been reported in conditions that promote the development of LH (Takase et al., 2005).

Interestingly, abnormal activation of the LC has been associated with the development of helpless behavior (Simson and Weiss, 1988; Vollmayr and Henn, 2003; Takase et al., 2005), and increased LC response to corticotropin-releasing factor (CRF) has been linked to depression (Bangasser et al., 2010). These findings, together with our result that LC is the only subcortical structure showing higher activity in the helpless mice compared to the resilient mice, strongly suggest a role of LC hyperactivity in the pathogenesis of stress-induced depression.

We also identified novel brain regions previously not associated with helplessness. For example, the OT, an area involved in odor processing as well as high cognitive functions including reward processing (Wesson and Wilson, 2011), and the Edinger–Westphal (EW) nucleus containing centrally projecting neurons implicated in stress adaptation (Kozicz et al., 2011), had decreased activation in the helpless mice compared to the resilient mice.

Finally, by taking advantage of the whole-brain activity data, which were generated with complementary methods for measuring neural activity in different species, we found that the brains of helpless animals are locked in a highly stereotypic pathological state, which may serve as an novel endophenotype of depression-like behavior (Hasler et al., 2004).

Our approach to identifying neuronal activity patterns related to LH is unique compared with previous studies in this field, in that it takes advantage of the recently developed unbiased single-cell resolution whole-brain mapping strategy (Kim et al., 2015). Indeed, this approach allowed us to identify a list of brain areas – many of which have not been reported thus far – as well as novel features of brain activity that might be critical for the expression of the stress-induced behavioral phenotypes. Future studies aimed at manipulating these identified neural changes are required for determining whether they are causally related to the expression of helplessness or resilience. Altogether, our findings provide novel insights into brain circuits underlying LH, a model of depression, which can guide future studies aimed at elucidating the specific roles of these regions in the pathophysiology of depression as well as serve as neural circuit-based targets for the development of novel therapeutics.

## Materials And Method

### Animals

Mice were group housed under a 12 h light/dark cycle (lights on: 9 a.m.–9 p.m.), and were separated into individual cages 24 h prior to experiments. Animals received standard pellet diet and water *ad libitum*. The *c-fosGFP* mice, which were described previously (Reijmers et al., 2007), were purchased from The Jackson Laboratory and were bred onto C57BL/6N background. Littermate male mice of 9–11 weeks-old were used.

Sprague-Dawley rats were purchased from Taconic Farms and allowed to acclimate to the animal facility for 2 weeks prior to experiments. Two rats were housed in each cage under a 12 h light/dark cycle (lights on: 7 a.m.–7 p.m.) with food and water *ad libitum*. Male rats of 3–5 months-old and 350–500 g in weight were used.

All procedures involving animals were approved by the Institutional Animal Care and Use Committees of Cold Spring Harbor Laboratory and Brookhaven National Laboratory.

### Behavioral Procedures

#### Mouse Study

The LH procedure in mice has been described previously (Chourbaji et al., 2005; Wang et al., 2014; Perova et al., 2015). Briefly, mice were first exposed to two induction sessions that were separated by 24 h. Each session consisted of 360 inescapable, uncontrollable electric foot shocks over a 60 min period. The shock intensity was set at 0.3 mA, shock durations were randomized between 1 and 3 s, and inter-shock-intervals (ITIs) were randomized between 1 and 15 s.

At 24 h after the second induction session, mice were subjected to a testing session. The testing, fully automated using Graphic State 3.0 software (Coulbourn Instruments), was performed in a shuttle box (35.5 cm × 18 cm × 30.5 cm; Coulbourn Instruments) equipped with an electrical grid floor, a door separating the two halves, and photocell detectors. The shuttle box was placed in a sound-attenuating chamber. Mice explored the shuttle box for 2 min, and behavioral performance was evaluated over 30 trials (trials 5–35) of escapable foot shocks (0.3 mA intensity, 10 s duration, with ITIs of 30 s). Each trial started with a 5 s cue light, followed by the foot shocks. The first five trials were not scored, because during these initial trials animals were learning the association between the light cue and the foot shock. When an animal shuttled to another compartment of the box during the 5 s cue light presentation (and therefore before the shock onset), avoidance was scored. If the animal shuttled during the 10 s shocks (i.e., escaped), escape latency was measured. Failure was recorded if no shuttling was made during the 10 s shock presentation. Shock was terminated if the animal shuttled to another side of the box (in case of escape) or at the end of the 10 s shock (in case of failure). Both the induction and testing sessions were conducted during the dark cycle.

Animals’ behavior was classified as being “resilient” or “helpless” based on their performance parameters in the LH testing session. A *k*-means (*k* = 2) cluster analysis was applied to the dataset collected from 144 mice that underwent LH procedure (**Figure 1**). We used the number of failures and escape latency as parameters for classification as these are the most commonly reported indices of helplessness (Abramson et al., 1978; Chourbaji et al., 2005; Wang et al., 2014; Perova et al., 2015). We further performed a linear discriminant analysis on our clustering results, with the number of failures and escape latency as predictor variables, to obtain classification equations for new cases: *R* = -4.63 + (5.67^∗^escape latency) + (-1.65^∗^failures), and LH = -23.24 + (3.67^∗^escape latency) + (0.53^∗^failures), where the escape latency and the number of failures define the classification score R (resilience) and LH. A mouse is classified as being resilient if *R* > LH, or helpless if LH > *R*. A higher classification score reflects a smaller squared Mahalanobis distance to the centroid of the corresponding group (Chourbaji et al., 2005; Wang et al., 2014; Perova et al., 2015).

#### Rat Study

The LH procedure in rats has been described previously (Li et al., 2011). Briefly, rats were exposed to one induction session consisting of 120 inescapable and uncontrollable foot-shocks over a 40 min period in an operant chamber (30.5 cm × 24.5 cm × 30.5 cm; Coulbourn Instruments) equipped with an electrical grid floor and fully automated by Graphic State software (Coulbourn Instruments). The shock intensity was 0.4 mA; shock durations and the ITIs were randomized between 5 and 15 s. The testing session was conducted 24 h following the induction and consisted of 15 trials of foot shocks, during which an illuminated lever was added to the chamber so that animals could terminate the foot shocks by pressing the lever. Animals that frequently escaped the foot shocks by lever pressing were classified as being resilient (≥10 lever presses), whereas those that had deficits in escaping were classified as being helpless (≤5 lever presses). For increased stringency, only lever presses occurring within the first 20 s of shock onset were counted. The experiments were conducted between 9 a.m. and 11 a.m.

### Whole-Brain Imaging and Data Acquisition

#### STPT Imaging on c-FosGFP Mice

Mice were transcardially perfused with saline and 4% paraformaldehyde (PFA) at 3 h after the testing session. The brains were further fixed in 4% PFA at 4°C overnight, followed by two more days in 0.1 M phosphate buffer (PB) with 0.1 M glycine at 4°C to reduce background autofluorescence. The brains were subsequently stored in 0.05 M PB at 4°C for up to 1 month until imaging. Detailed information about STPT imaging and related analysis procedures is described previously (Ragan et al., 2012; Kim et al., 2015). Briefly, brain was embedded in 4% oxidized agarose and crosslinked by sodium borohydrate. The embedded brain was placed on the motorized stage in tissuecyte 1000 (Tissuevision) and the whole-brain was imaged at a resolution of 1 μm at the x–y plane for a series of 280 z sections with 50 μm inter-section-interval. Both the signal from the green channel (c-FosGFP signal) and that from the red channel (background) were simultaneously acquired, and the latter was used to subtract background from the green channel to enhance signal to noise ratio. Automatic cFos-GFP signal detection in the background subtracted images was achieved by convolutional neural network, a type of machine learning algorithm, that was previously trained and validated to reliably detect cFos-GFP signal throughout the entire brain (Kim et al., 2015). The detection method provides consistent cell counting in most brain regions except relatively poor detection in heavily myelinated brain regions such as caudal OT area (Kim et al., 2015).

Image registration (Elastix) was used to map detected signals onto a reference STPT brain as described previously (Kim et al., 2015).

#### 18FDG-PET Study on Rats

Out of the 24 rats tested in this study, 37.5% became helpless (*n* = 9), and 37.5% were resilient (*n* = 9). The animals having an intermediate number of lever presses and/or test completion time were excluded from further analysis (41.7%; *n* = 6). There were three animals (one per group) that were excluded due to poor i.p. injection of the radiotracer. On the morning of the imaging experiment, animals were transported from the animal facility into a quiet room adjacent to the behavioral testing room and after a 30-min wait period in their home cage, animals were transferred to operant chambers and the LH testing protocols ensued. Animals were then placed back to their home cage, transported to the PET facility, and allowed an additional 30 min of acclimation prior to the injection of ^18^FDG radiotracer. To maximize the transport of radioactively labeled glucose into the brain, all animals were food deprived for a total of 4 h prior to radiotracer administration. We used previously established procedure for PET scan and imaging analysis (Mirrione et al., 2014).

### Statistical Analysis

#### Identifying Brain Regional Differences

The *c-fosGFP* mice used for imaging were exclusively chosen in pairs, each pair containing a resilient and a helpless mouse that had undergone the entire experimental procedure in parallel. We performed pairwise statistics to identify differentially activated brain regions in helpless versus resilient mice. This strategy has higher statistical power than conventional student *t*-test and is less affected by potential fluctuations in c-FosGFP expression that are caused by non-specific factors, such as the time and date when mice were sacrificed.

Briefly, we measured the effect that the experimental conditions had on the observed cell counts (that is, the number of GFP^+^ neurons) *Y* with a generalized linear model. We assumed the conditional distribution of *Y* could be modeled with a negative binomial distribution, a conventional choice for datasets of over-dispersed integer counts. The experimental condition was represented by a binary categorical variable *G*, which indicated the group identity (helpless versus resilient) of the sample. Information about the pairing in samples was represented by a blocking variable *B*, a categorical variable with a number of states corresponding to the number of pairs in the samples. For each brain region, we obtained the effect of *G* on *Y* while controlling for the confounding effect of *B* by looking at the significance of the type I coefficient of *G*; that is, we modeled the conditional mean of *Y* as a linear combination of *B* and *G*, and took the *P*-value from the sequentially added *G* term. Once the *P*-values from different anatomical regions were determined, they were globally adjusted across every region to correct for multiple comparisons via Benjamini and Hochberg’s false discovery rate procedure.

#### Correlation Between Brain Samples

Signals (c-FosGFP-expressing cells, or PET signal) in anatomic regions across the entire brain from each individual animal were used to calculate the Pearson correlation coefficient between individual animals. We used a bootstrap test (in *R* programming environment) to compare the correlations among the LH mice with those among the resilient (R) mice. The null hypothesis is that there is no difference between LH and R groups. Specifically, we randomly sampled (with replacement) n1 mice (n1 = 11 and 8 for the c-FosGFP and rat PET dataset, respectively) from the combined datasets (LH and R animals altogether) and assigned them to the “LH^null^” group, and similarly sampled and assigned n2 mice (n2 = 11 and 8 for the c-FosGFP and rat PET dataset, respectively) to the “R^null^” group. We then computed the Pearson correlation coefficients between individuals in each of the two groups, and took the difference between group means (Correlation^LHnull^ – Correlation^Rnull^). This process was repeated 100,000 times and the resulting values were used to build up a null distribution. Finally, we examined where in this distribution fell the difference between the actual LH and R groups in their mean Pearson correlation coefficients (Correlation^LH^ – Correlation^R^), hence obtaining a bootstrap *P*-value. The two-sided *P*-values were reported for all comparisons.

### Supplementary Methods

#### Immunohistochemistry in Mice

Immunohistochemistry was performed following previously described procedures (Wang et al., 2014). Briefly, mice were deeply anaesthesized and transcardially perfused with PBS, followed by 4% PFA in PBS. Brains were extracted and further fixed in 4% PFA overnight at 4°C followed by cryoprotection in a 30% PBS-buffered sucrose solution for 36 h. Coronal sections of 50 μm were cut using a freezing microtome (Leica SM 2010R). Sections were first washed in PBS (3 min × 10 min) and then blocked in 5% normal goat serum in PBST for 60 min at room temperature, followed by incubation with primary anti-c-fos antibody (rabbit, Santa Cruz Biotechnology, 1:5000) overnight at 4°C. Sections were then washed with PBS (3 min × 10 min) and incubated with fluorescent secondary antibody at room temperature for 1 h. After washing with PBS (3 min × 10 min), sections were mounted onto slides with Fluoromount-G (Beckman Coulter). Images were taken using an Olympus BX51 epifluorescent microscope.

## Author Contributions

BL, PO, FH, YK, ZP, and MM designed the study. YK, ZP, and MM conducted experiments. KP, SS, YK, ZP, and MM analyzed data. YK and BL wrote the paper with inputs from all authors.

## Conflict of Interest Statement

The authors declare that the research was conducted in the absence of any commercial or financial relationships that could be construed as a potential conflict of interest.

## Supplementary material

TABLE S1 **A full list of brain areas showing differential activity in helpless versus resilient animals.** Cortical areas include detailed layer information. Positive z scores indicate higher activity in resilient group than helpless group, whereas negative z scores (highlighted in red) indicate higher activity in helpless group than resilient group.

TABLE S2 **The full raw dataset for cfos-GFP^+^ cell counting in each mouse. Eleven pairs of mice, each containing one helpless and one resilient mice, were imaged and analyzed.** The table contains the number of detected cFos-GFP^+^ cells in each anatomical area across the whole-brain.

FIGURE S1 **Endogenous c-Fos expression in mice showing helpless or resilient behavior. (A)** Representative images of c-Fos expression detected by immunohistochemistry from a helpless (left) and a resilient (middle) mouse (*n* = 5 mice for each group). PL, prelimbic area; ILA, infralimbic area; fa, corpus calossum anterior forceps. Right: higher magnification images of the boxed areas in the left and middle panels. **(B–E)** Increased c-Fos expression was observed in different regions of the mPFC in resilient mice compared with helpless mice. **(B)** Overall c-Fos expression. PL, *T*_(8)_ = 3.349, ^∗^*P* < 0.05; ILA, *T*_(8)_ = 3.076, ^∗^*P* < 0.05. **(C,D)** c-Fos expression in different layers. **(C)** Layer 1 (L1). PL_L1, *T*_(8)_ = 3.702, ^∗∗^*P* < 0.01; ILA_L1, *T*_(8)_ = 1.098, *P* = 0.3. **(D)** Layers 2 and 3 (L2/3). PL_L2/3, *T*_(8)_ = 2.626, ^∗^*P* < 0.05; ILA_L2/3, *T*_(8)_ = 1.965, *P* = 0.085. **(E)** Layers 5 and 6 (L5/6). PL_L5/6, *T*_(8)_ = 2.898, ^∗^*P* < 0.05; ILA_L5/6, *T*_(8)_ = 4.054, ^∗∗^*P* < 0.01. Unpaired *t*-test.
