# Supplementary figures and images for: Whole-Brain Mapping of Neuronal Activity in the Learned Helplessness Model of Depression
Source: Front Neural Circuits. 2016 Feb 3;10:3. doi: 10.3389/fncir.2016.00003 (PMC4737884; doi:10.3389/fncir.2016.00003)

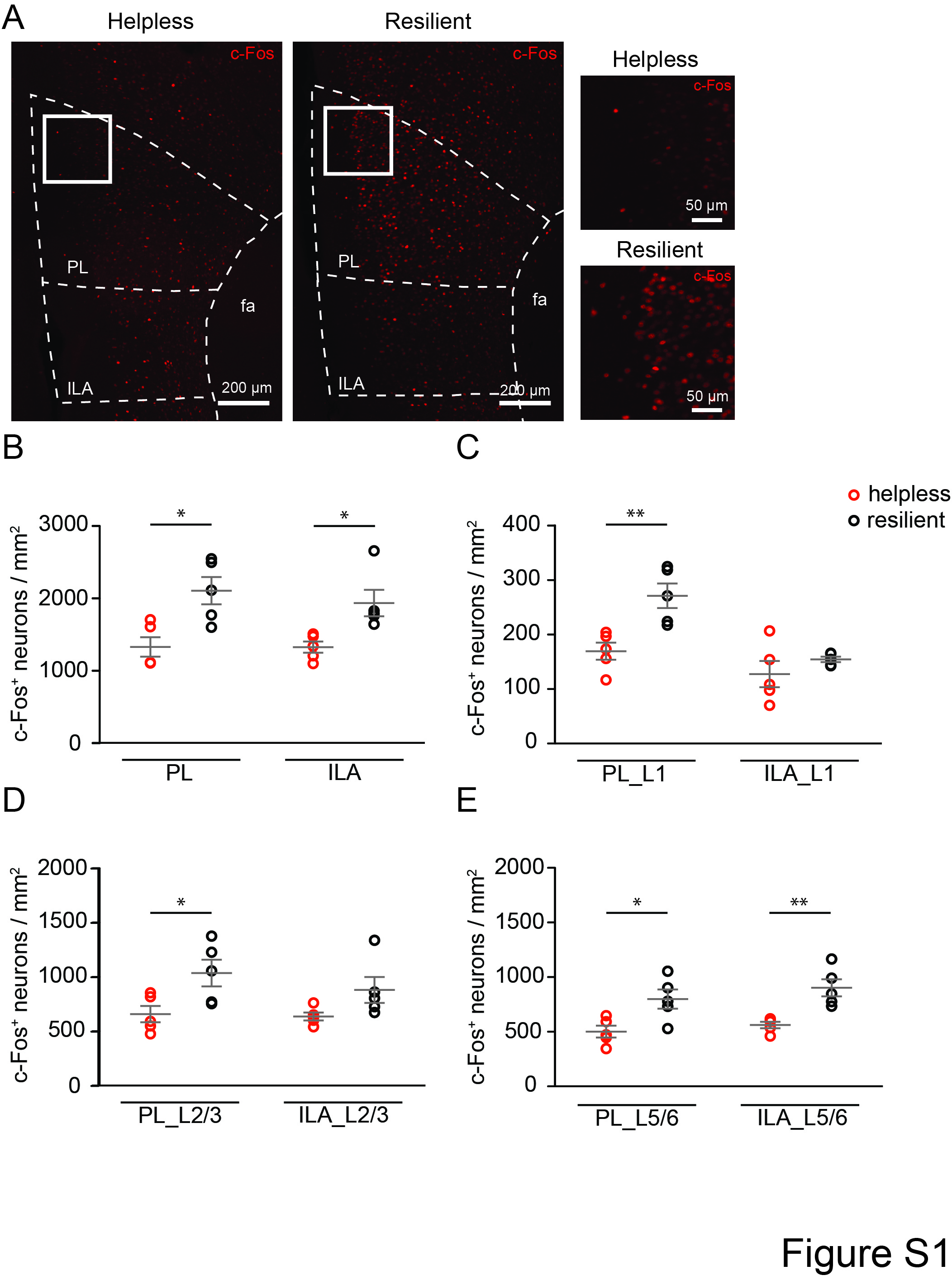

Supplement: FIGURE S1 — Endogenous c-Fos expression in mice showing helpless or resilient behavior. (A) Representative images of c-Fos expression detected by immunohistochemistry from a helpless (left) and a resilient (middle) mouse (n = 5 mice for each group). PL, prelimbic area; ILA, infralimbic area; fa, corpus calossum anterior forceps. Right: higher magnification images of the boxed areas in the left and middle panels. (B–E) Increased c-Fos expression was observed in different regions of the mPFC in resilient mice compared with helpless mice. (B) Overall c-Fos expression. PL, T(8) = 3.349, ∗P < 0.05; ILA, T(8) = 3.076, ∗P < 0.05. (C,D) c-Fos expression in different layers. (C) Layer 1 (L1). PL_L1, T(8) = 3.702, ∗∗P < 0.01; ILA_L1, T(8) = 1.098, P = 0.3. (D) Layers 2 and 3 (L2/3). PL_L2/3, T(8) = 2.626, ∗P < 0.05; ILA_L2/3, T(8) = 1.965, P = 0.085. (E) Layers 5 and 6 (L5/6). PL_L5/6, T(8) = 2.898, ∗P < 0.05; ILA_L5/6, T(8) = 4.054, ∗∗P < 0.01. Unpaired t-test. [file Image_1.JPEG]
